# The cancer nursing workforce in Australia: a national survey exploring determinants of job satisfaction

**DOI:** 10.1186/s12912-023-01629-7

**Published:** 2023-12-06

**Authors:** Natalie Bradford, Elizabeth Moore, Karen Taylor, Olivia Cook, Lucy Gent, Theresa Beane, Natalie Williams, Kimberly Alexander, Erin Pitt, Jemma Still, Cameron Wellard, Gemma McErlean, Deborah Kirk, Leanne Monterosso, Alexandra McCarthy, Zerina Lokmic-Tomkins, Jessica Balson, Priscilla Gates, the CNSA Research Standing Committee, the CNSA Board of Directors, Meredith Cummings, Anne Mellon, Diane Davey, Sue Schnoonbeek, Gabby Vicar, Kate White

**Affiliations:** 1 Cancer and Palliative Care Outcomes Centre and School of Nursing, Queensland University of Technology, QLD, Brisbane, QLD Australia; 2 Cancer Nurses Society of Australia, Gabbadah, WA Australia; 3 School of Public Health and Preventive Medicine, Monash University, Melbourne, VIC Australia; 4 Cancer Network WA, Perth, WA Australia; 5 McGrath Foundation – Level 1, 32 Walker St North Sydney, Sydney, NSW Australia; 6 Monash Nursing and Midwifery, Monash University, Melbourne, VIC Australia; 7 Sir Charles Gairdner Osborne Park Hospitals Health Care Group, Perth, WA Australia; 8 Edith Cowan University, Perth, WA Australia; 9 Hervey Bay Hospital, Queensland Health, Pialba, QLD Australia; 10 King Edward Memorial Hospital, Perth, WA Australia; 11 School of Nursing, University of Wollongong, Wollongong, NSW Australia; 12 Notre Dame University, Perth, WA Australia; 13 The University of Queensland, Brisbane, QLD Australia; 14 Peter MacCallum Cancer Centre, Melbourne, VIC Australia; 15 Deakin University, Melbourne, VIC Australia

**Keywords:** Workforce, Job satisfaction, Workload, Cross sectional studies, Leadership

## Abstract

**Background:**

To maintain and improve the quality of the cancer nursing workforce, it is crucial to understand the factors that influence retention and job satisfaction. We aimed to investigate the characteristics of cancer nurses in Australia and identify predictors of job satisfaction.

**Methods:**

We analysed data from an anonymous cross-sectional survey distributed through the Cancer Nurses Society Australia membership and social media platforms from October 2021 to February 2022. The survey was compared to national nursing registration data. Data were analysed with non-parametric tests, and a stepwise, linear regression model was developed to best predict job satisfaction.

**Results:**

Responses were received from 930 cancer nurses. Most respondents (85%) described themselves as experienced nurses, and more than half had post-graduate qualifications. We identified individual, organizational, and systemic factors that contribute to job satisfaction and can impact in workforce shortages. The findings include strategies to address and prioritize workforce challenges. There were 89 different titles for advanced practice nursing roles. Managing high workload was a reported challenge by 88%. Intention to stay less than 10 years was reported by nearly 60%; this was significantly correlated with job satisfaction and age. Significantly higher scores for job satisfaction were associated with those who had career progression opportunities, career development opportunities, adequate peer support and a clearly defined scope of role. Conversely, job satisfaction scores decreased the more people agreed there was a lack of leadership and they had insufficient resources to provide quality care.

**Conclusion:**

Cancer nurses are critical to the delivery of cancer care however, the workforce faces multiple challenges. This study provides an understanding of the Australian cancer nursing workforce characteristics, their roles and activities, and highlights important considerations for retaining nurses in the profession.

**Supplementary Information:**

The online version contains supplementary material available at 10.1186/s12912-023-01629-7.

## Background

Over the next 50 years, the incidence and prevalence of cancer are expected to continue increasing worldwide driven by population growth, aging and improved diagnosis and reporting [1]. This trend highlights the need for a strong and capable workforce to deliver quality care to patients. However, the global nursing workforce is challenged by low recruitment and retention, and high turnover with nurses leaving the profession, which makes it difficult to meet the demand for services [2–4]. Numerous studies have shown the correlation between appropriate staffing on outcomes including length of stay, unplanned hospital admissions and mortality rates [5–8]. The shortage of cancer nurses directly threatens decades of progress made in improving outcomes for patients with cancer and is imperative to address.

Cancer nursing is a highly specialised field and has rapidly evolved over the past decade with novel anti-cancer therapeutics that have changed the landscape of care, education and management of patients [9]. The expert cancer nurse possesses the ability to adapt and fluidly apply knowledge and experience to new and unexpected situations in the management of patient care [10]. Essential components of quality cancer nursing care include delivering person-centred and integrated care across the continuum of cancer care with great skill in communication and care coordination [11]. The knowledge, skills and experience of expert cancer nurses are not easily replaced highlighting the need to invest in the workforce.

Job satisfaction is recognised as an important determinant of both intention to stay and actual turnover in the nursing workforce [12, 13]. Given this, understanding the determinants of job satisfaction is crucial to addressing shortages in the cancer nursing workforce [14]. In their seminal theoretical work, Irvine et.al investigated the causal relationship between job satisfaction, intention to leave and nurse turnover and proposed work content and work environment had a stronger relationship with job satisfaction compared to economic or individual characteristics [15]. This theoretical work helps with understanding relationships between concepts, although given the social and economic changes since this work was published, a contemporary exploration is warranted.

Despite the importance of understanding the cancer nursing workforce, there is a lack of contemporary data, even in high-income countries, that characterize the demographic and geographical features or explore workforce issues at the population level [16–18]. With retention of nurses a near universal challenge across the globe, understanding these factors is critical in order to predict needs and develop strategies to intervene and strengthen the workforce [19, 20]. We thus aimed to examine the cancer nursing workforce in Australia and answer the following research questions:

1. What are the characteristics of cancer nurses in Australia?
  Where do they live and work, what are their qualifications and how long have they been practicing?
2. What activities are cancer nurses involved in?
3. What are the challenges to the cancer nursing workforce?
4. What individual, organisational and systems level variables predict cancer nurses job satisfaction?

Our objectives were to:

1. Investigate the demographic and geographical distribution of cancer nurses in the country
2. Identify predictors of job satisfaction among cancer nurses

## Methods

### Study design and context

This was a cross-sectional, national survey conducted by the Cancer Nurses Society of Australia (CNSA) and compared with national nurse registration data. The CNSA, founded in 1998, is the peak professional body for cancer nursing in Australia, representing more than 1500 cancer nurses across the country. The CNSA’s mission is to promote excellence in cancer care [21].

### Sample and setting

The survey was distributed in October 2021 via email to members of the CNSA, promoted in the CNSA newsletter, as well as through other professional networks and on social media platforms such as Facebook™ and Twitter™. The survey remained open until February 2022. In addition, nurse registration data for the year 2021 was obtained from the Australian Health Workforce data [22].

### Data collection/ measures

A 68-item survey was developed by the CNSA's Research Standing Committee for this study and was informed by relevant literature, incorporating questions from other published nursing workforce evaluations [23, 24], items from the Nursing Work Index-Revised [25], and in consultation with key stakeholders. The survey is available in the Supplementary file. Domains of job satisfaction included satisfaction with the work and workplace, peers, pay, opportunities for career progression and supervision as well as an overarching question where respondents were asked to rate their job satisfaction on a continuous scale from 0–100 with higher values indicating higher satisfaction. To explore variables at the individual level, the survey included demographic questions such as age, gender, postcode, years of nursing and cancer nursing experience, qualifications, involvement in professional organizations and intention to stay in the nursing workforce. For variables at the organisational level, respondents were asked to provide information on their nursing role, type of facility, full-time equivalent worked, permanency of their position, cancer specialty area, and their usual work activities. Statements about work environments from the Nurse Work Index Revised [25] at the organisational and systems level including rates of pay, satisfaction with pay, and scope of practice and were measured for level of agreement on a five-point Likert scale. There were also free-text options for participants to provide further information about workforce issues and suggestions for initiatives to support the cancer nursing workforce (to be reported elsewhere).

Face validity was determined through pilot testing among a sample of nine cancer nurses who represented a diverse range of nurses working in different settings. Wording was modified based on the feedback received to improve flow. The survey was electronically distributed and managed using the REDCap [26] platform, and included branching logic to reduce survey fatigue. Participants were provided with a participant information sheet and completion of the survey implied consent. The responses did not ask for personal identifiers (names, birthdates) or the names of workplace and were anonymous.

For comparison and to understand the representativeness of the survey data, Health Workforce data from the Australian Department of Health [22] was obtained. These data were collected in 2021 as part of professional registration and provided comparative demographic data for all nurses in Australia as well as one question regarding intention to stay in the workforce- we also included this question in our CNSA survey adding the term cancer ‘*how long do you intend to stay in the* [cancer]* nursing workforce?’.*

The study was ethically approved by Monash University Human Research Ethics Committee and the Queensland University of Technology Research Governance and Integrity (Project ID: 30,474, Project ID 6544).

### Data analysis

Demographic data were compared between the 2021 Health Workforce survey and the CNSA survey respondents to determine the representativeness of our findings and to describe the national characteristics of the cancer nursing workforce. Data were exported to Excel and analysed using statistical software (Stata IC/16.0) to provide descriptive statistics such as proportions, medians and interquartile ranges that describe the profile of cancer nurses in Australia. We grouped free text responses for nursing roles into categories for analysis based upon classifications in the Australian Nurses Award 2020 with an additional category for Advanced Practice Nurses. Nurses who responded they held a specialist cancer nursing role were grouped into this category. Data were coded by one author and checked by a second. Proportions for individual questions were calculated from the number of respondents for each question. Chi Square test were used to explore differences in the CNSA sample and AHPRA 2021 data. Given job satisfaction scores were not normally distributed, non-parametric Kruskal–Wallis rank test or Mann–Whitney U rank-sum test were used to explore differences in job satisfaction score across categorical variables for individual (e.g. age, years of experience, qualifications), organisational (e.g. Cancer speciality activities involved in), and systems level variables (rates of pay, resources to provide care). A stepwise model building approach was used to develop a final parsimonious linear regression model that best predicted job satisfaction score [27]. Predictor variable were dichotomized where possible. Firstly, bivariate regression models were run to identify significant (*P* < = 0.05) or near-significant (*P*< 0.20) associations between each predictor and job satisfaction. Significant predictors were then added to the linear regression model one at a time to establish the effect of each variable on job satisfaction and the other predictors. Non-significant predictor variables from bivariate modelling were then re-added to test their effect on the overall model. The final parsimonious linear regression model was identified with the inclusion of predictor variables that explained the most variation in job satisfaction score [27].

## Results

### Characteristics of cancer nurses from the CNSA survey and AHPRA 2021 workforce data

Responses were received from 930 cancer nurses, with 858 providing demographic data. Of these, 507 (77%) were members of the CNSA (Table 1). Given the CNSA membership was approximately 1500 at the time of the survey, and 7202 nurses indicated they were cancer nurses in the AHPRA 2021 Workforce survey, our response rate was approximately 34% of CNSA members and 13% for all cancer nurses.

**Table 1:** Sample characteristics of the CNSA sample compared with AHPRA 2021 workforce data

Variables		CNSA 2021 Sample		AHPRA 2021 Data		*P* value
		**N**	**%**	**N**	**%**	
*Gender*		*846*		*7200*		
	Male	43	5.1%	523	7.3%	-
	Female	800	94.6%	6677	92.7%	
	Non-binary	3	0.4%	-	-	
*Age group*		*858*		*7200*		
	20–34	163	19.0%	2679	37.2%	< 0.001
	35–44	226	26.3%	1717	23.8%	
	45–54	233	27.2%	1539	21.4%	
	55–64	216	25.2%	1096	15.2%	
	65 +	20	2.3%	169	2.3%	
*State*		*857*		*7187*		
	New South Wales	179	20.9%	1875	26.1%	< 0.001
	Victoria	243	28.4%	2068	28.8%	
	Queensland	200	23.3%	1728	24.0%	
	South Australia	70	8.2%	497	6.9%	
	Western Australia	117	13.7%	621	8.6%	
	Tasmania	37	4.3%	188	2.6%	
	Northern Territory	4	0.5%	38	0.5%	
	Australian Capital Territory	7	0.8%	172	2.4%	
*Remoteness*		*817*		*7201*		
	Major cities	557	68.2%	5823	80.9%	< 0.001
	Regional	221	27.1%	1342	18.6%	
	Remote	39	4.8%	36	0.5%	
*Main Role*		*807*		*7202*		
	Clinician	601	74.5%	6787	94.2%	< 0.001
	Administrator	75	9.3%	174	2.4%	
	Teacher or educator	53	6.6%	72	1.0%	
	Researcher	32	4.0%	131	1.8%	
	Other	46	5.7%	38	0.5%	
*Intention to stay in (cancer)*^a^* nursing*		*765*		*5905*		
	Less than 5 years	225	29.4%	1145	19.4%	< 0.001
	5–9 years	220	28.8%	1315	22.3%	
	10–19 years	164	21.4%	2093	35.4%	
	20 years +	153	20.0%	1352	22.9%	

^a^AHPRA survey asks about intention to stay in nursing

Demographic details from the CNSA sample were compared with the AHPRA 2021 data. The CNSA sample was significantly older, with fewer nurses represented in the 20–34-year age group (19% vs 37%, *p* = < 0.001). Additionally, the CNSA sample had a higher representation of nurses from less-populated states and regional areas compared to the AHPRA data, and a higher proportion of nurses working in non-clinical areas, such as administration, teaching, or research. Furthermore, the CNSA sample was found to be more likely to leave the workforce within the next five years (29% vs 19%, *P* = < 0.001) which may be attributed to CNSA members being a comparatively older cohort of nurses.

### Cancer nursing experience and qualifications

Most respondents (85%) described themselves as experienced nurses, who predominantly provided clinical cancer nursing care to patients; 35% had 10–19 years of experience and a further 30% had over 20 years of experience working in cancer care. More than 55% had postgraduate qualifications, with 62% having a cancer-related qualification, most commonly a graduate certificate or diploma. Another 27% reported they planned to obtain cancer-related qualification. A wide variety of roles were reported across clinical care, education, administration, and research (Table 2). Significant differences were found when data were stratified by state of residence; nurses in Tasmania were more likely to reside in regional locations, and to have recently (≤ 3 years) graduated. Nurses from Victoria and New South Wales, the most populous states of Australia, were more likely to have cancer-related post-graduate qualifications.

**Table 2:** Experience, qualifications, and role

**Variables**		**N**	**%**
*Years of nursing experience*		*858*	
	Less than 5 years	68	7.9%
	5 to 9 years	109	12.7%
	10 to19 years	243	28.3%
	20 + years	438	51.0%
*Years of cancer nursing experience*		*857*	
	Less than 5 years	154	18.0%
	5 to 9 years	149	17.4%
	10 to19 years	297	34.7%
	20 + years	257	30.0%
*Post-graduate qualification*		*854*	
	Yes	474	55.5%
	No	380	44.5%
*Cancer-related qualification*		*885*	
	Yes	552	62.4%
	No	333	37.6%
*Self-nominated *^a^*EdCaN professional development model level of competency*		*669*	
	Can demonstrate core capabilities in cancer care	20	3.0%
	Can apply core capabilities at an advanced level	41	6.1%
	Provides specialist cancer care adhering to competency standards	420	62.8%
	Practices at an advanced level applying competency standards	188	28.1%
*Nursing role*		*802*	
	Advanced Practice Nurse (clinical roles)	259	32.3%
	Registered nurse	211	26.3%
	Clinical Nurse /Trials Nurse	114	14.2%
	Nurse Unit Manager	69	8.6%
	Nurse Educator	53	6.6%
	Researcher/ Academic	14	1.7%
	Nurse Practitioner	30	3.7%
	Director of Nursing	6	0.7%
	Other	46	5.7%
*Average number of days worked per week*			
	5 days per week (full time)	296	34.4%
	3–4 days per week	437	50.9%
	1–2 days per week	67	7.8%
	Not stated	58	6.8%

^a^EdCaN professional development model for specialist cancer nurses [28]

### Cancer nursing roles and activities

Respondents were asked to self-describe the title of role in cancer nursing returning 89 unique role titles across specialist nurse roles. Some role titles are explained by different disease types, however many, particularly for Advanced Practice Nurses, are ambiguous and widely varied across institutions and geographical locations. The terms ‘Cancer’ and ‘Clinical’ are often used interchangeably, for example, ‘Cancer Nurse Specialist’ and ‘Clinical Nurse Specialist’; ‘Cancer Nurse Consultant’ and ‘Clinical Nurse Consultant’. The impact of non-standardized nomenclature on the clarity and understanding of both patients and other health professionals is unknown. Respondents reported working across different types of cancer facilities, including specialist cancer centres that provide multidisciplinary services through to primary care settings including general practice and community-based services.

Most respondents reported working across multiple disease types and specialties, including rare cancers as well as pediatric, adolescent, and young adult cancer. A variety of activities were reported by most nurses such as delivery of patient education (77%), outpatient care (66%), staff education (59%), treatment and supportive care (55%). There were fewer nurses involved in management (24%), research (22%), radiotherapy (19%), surgical care (12%) and home care (5%) (Table 3).

**Table 3:** Oncology specialty, disease type and activities

Variables	N	%
*Main cancer(s) specialty*	*781*^a^	
Medical Oncology	476	60.9%
Haematological Oncology	363	46.5%
Radiation Oncology	183	23.4%
Palliative Care	135	17.3%
Pediatric / Adolescent Young Adult Oncology	113	14.5%
Surgical Oncology	94	12.0%
Community Care	35	4.5%
Other	66	8.5%
*Main disease type*	*774*^a^	
Haematology	463	59.8%
Breast	458	59.2%
Lung Cancer	426	55.0%
Lower GI	408	52.7%
Prostate	395	51.0%
Upper GI	388	50.1%
Melanoma	372	48.1%
Gynaecological	359	46.4%
Brain/Central Nervous System	338	43.7%
Urogenital	316	40.8%
Neuroendocrine	246	31.8%
Sarcoma	241	31.1%
Rare cancers (including paediatric)	160	20.7%
Other	77	9.9%
*Activities most involved in*	*776*^a^	
Patient education	599	77.2%
Outpatient care	515	66.4%
Staff education	459	59.1%
Supportive care (e.g., transfusions, manage infections)	442	57.0%
Care coordination	414	53.4%
Chemotherapy / Immunotherapy administration	407	52.4%
Inpatient care	367	47.3%
Palliative care	262	33.8%
Management	185	23.8%
Research	174	22.4%
Radiotherapy	152	19.6%
Surgical care	95	12.2%
Homecare	41	5.3%

^a^Respondents could choose more than one category

### Workplace challenges to the cancer nursing workforce

Managing a high workload was the most frequently reported challenge (88% of respondents) with information overload (53%), insufficient resources (41%) and lack of leadership (39%) contributing to workplace challenges (Table 4). Nurses reported variance in opportunities for career progression, professional development, full use of the extent of their knowledge, and having a clearly defined role and peer support, further contributed to challenges. Selected variables were included in bivariate and multivariate analysis to predict job satisfaction as reported below.

**Table 4:** Challenges to the cancer nursing workforce

What are the challenges to the cancer nursing workforce in your workplace?	Agree or strongly agree	
	N	%
Managing high workload (*n* = 708)	623	88%
Information overload (*n* = 702)	373	53%
Integrating digital health technologies, e.g. telehealth, electronic medical records (*n* = 695)	310	44%
Insufficient resources to provide quality care (*n* = 704)	287	41%
Poor clinical supervision or mentorship (*n* = 704)	281	40%
Lack of leadership in the workplace to support the workforce (*n* = 705)	277	39%
Lack of opportunities for career progression (*n* = 703)	263	37%
Lack of training and education opportunities (*n* = 703)	246	35%
Lack of clarity about roles/performance expectations (*n* = 703)	216	31%
Ineffective interagency collaboration (*n* = 699)	217	31%
Low motivation of staff to provide quality care (*n* = 704)	160	23%

Job satisfaction and intention to stay

The median score reported for job satisfaction was 75/100 (IQR 65, 88). Those who reported job satisfaction ≤ 50/100 were more likely (*P* = 0.011) to report intention to stay less than five years. Age was significantly correlated with both job satisfaction (*P* = 0.003) and intention to stay in cancer nursing (< 0.001), respondent in the older age categories reported higher job satisfaction compared to younger age groups but also had the highest intention to stay less than 5 years. Those in the youngest age category (20–34 years) reported the lowest job satisfaction and while 38% indicated they intended to stay in the workforce more than 20 years, 26% signalled their intention to stay less than 5 years (Table 5).

**Table 5:** Bivariate analysis of age group with satisfaction with current job (scale 0–100) and intention to stay

	**Age Group**					*P* value
	20–34	35–44	45–54	55–64	65 +	
***Job satisfaction Median; (IQR)***						
	71; (60–81)	76; (65–86)	75; (60–85)	80; (70–90)	82; (73–95)	0.003
***Intention to stay N (%)***						
5 years or less	38 (25.9)	31 (15.7)	41 (19.9)	99 (51.3)	16 (94.1)	< 0.001
6–10 years	34 (23.1)	35 (17.7)	64 (31.1)	85 (44.0)	1 (5.9)	
11–20 years	19 (12.9)	54 (27.3)	85 (41.3)	6 (3.1)	0	
> 20 years	56 (38.1)	78 (39.4)	16 (7.8)	3 (1.6)	0	

Table 6 further explores differences in average job satisfaction scores across characteristics of the sample. There were minimal differences in satisfaction across the states and territories of Australia (data not shown). The highest rates of job satisfaction were reported by those working in Primary Care (med 85 (IQR 71–90) and the lowest for those in a cancer unit (med 73 (IQR 60.5–85)) (*P* = 0.004). Those in Registered Nurse and Researcher/Academic positions had the lowest and highest median job satisfactions scores, respectively (med 73 (IQR 59–85); med 83 (IQR 75–86)) (*P* = 0.082). Nurses with more than 20 years of nursing experience (med 78 (IQR 65–90)) and 5 years or less (med 80 (IQR 68–88)), reported the highest levels of job satisfaction (*P* = 0.002), as did those aged 55 years and older (55–64 years: med 80 (IQR 70–90); 65 + : med 82 (IQR 73–95)) (*P* = 0.003). Those who intended to stay in the nursing workforce 5 years or less had the lowest median levels of job satisfaction (med 72 (IQR 50–83); *P* = 0.001). Job satisfaction scores increased the more nurses agreed they had professional development opportunities (*P* = 0.001), career development opportunities (*P* = 0.001), adequate peer support (*P* = 0.001) and a clearly defined scope of role (*P* = 0.001). Conversely, job satisfaction scores decreased the more people agreed there was a lack of leadership (*P* = 0.001) and they had insufficient resources to provide quality care (*P* = 0.001).

**Table 6:** Bivariate analysis of satisfaction with current job (scale 0–100) with key sample characteristics

Variable	Median job satisfaction (IQR)	*P*-value
*Type of facility*^a^		
Cancer Centre	76 (67–89)	0.004
Cancer Service	75 (59–85)	
Cancer Unit	73 (60.5–84)	
Primary Care	85 (71–90)	
Multiple facilities	75 (68–85)	
Other	80.5 (72–90)	
*Years of nursing experience*		
Less than 5 years	80 (68–88)	0.002
5–9 years	70.5 (55–80)	
10–19 years	75 (64–86)	
20 or more years	78 (65–90)	
*Main role*		
Advanced Practice Nurse	77 (67.5–90)	0.082
Registered Nurse	73 (59–85)	
Clinical Nurse /Trials Nurse	75 (62–85)	
Nurse Unit Manager	79 (62–86)	
Nurse Educator	72 (57–80)	
Researcher/Academic	83 (75–86)	
Nurse Practitioner	83 (70–91)	
Director of Nursing	75 (73–99)	
Other	79 (65.5–90)	
*Average number of days worked per week*		
5 days per week (1.0FTE)	75 (64–89)	0.395
More than 3/Less than 5 days per week (0.7–0.9FTE)	77 (65–90)	
2–3 days per week (0.4–0.6FTE)	75 (66–85)	
Less than 2 days per week (0.1–0.3FTE)	71 (63–80)	
*Have Professional Development opportunities*		
Strongly Disagree	35.5 (19–67)	0.001
Disagree	68 (50–75)	
Neutral	70 (58–78)	
Agree	75 (66–85)	
Strongly Agree	85 (74–91)	
*Have career progression opportunities*		
Strongly Disagree	50 (20–73)	0.001
Disagree	70 (50–78)	
Neutral	72 (61.5–80)	
Agree	78 (70–86)	
Strongly Agree	90 (78–93)	
*Use of full extent of knowledge and skills*		
None of the time	22 (0–50)	0.001
Occasionally	63 (39.5–75)	
Often	75 (65–85)	
Most of the time	80 (70–90)	
*Intention to stay in cancer nursing*		
Less than 5 years	72 (50–83)	0.001
5- 9 years	80 (67–90)	
10–19 years	78 (70–89)	
20 + years	75 (67–85)	
*Age group in years*		
20–34	71 (60–81)	0.003
35–44	76 (65–86)	
45–54	75 (59.5–85)	
55–64	80 (70–90)	
65 +	82 (73–95)	
*CNSA Member*		
No	73 (63–85)	0.008
Yes	77.5 (67–89)	
*Satisfied with level of pay*		
No	72 (60–81)	< 0.001
Yes	80 (70–90)	
*Have a clearly defined role*		
Strongly Disagree	73 (21–93)	0.001
Disagree	71 (50–80)	
Neutral	70 (60–80)	
Agree	75 (65–85)	
Strongly Agree	82 (70–90)	
*Have adequate peer support*		
Strongly Disagree	33.5 (20.5–69)	0.001
Disagree	60 (34–72)	
Neutral	70.5 (62–80)	
Agree	75 (68–85)	
Strongly Agree	89 (75–93)	
*Have insufficient resources to provide quality care*		
Strongly Disagree	89.5 (76–98)	0.001
Disagree	83 (72–90)	
Neutral	75 (65–85)	
Agree	74 (62–81)	
Strongly Agree	70 (40–75)	
*Lack leadership*		
Strongly Disagree	90 (83–98)	0.001
Disagree	80 (72–90)	
Neutral	75 (66–85)	
Agree	72 (60–81)	
Strongly Agree	63 (31–75)	

^a^ Type of facility defined as:

• Cancer Centre: Provides specialised, multidisciplinary service and specialised interventions to manage common and rare cancers. Can provide outreach support

• Cancer unit: Provides a multidisciplinary service to manage most common cancers. Can provide outreach support

• Cancer Service: Consists of single service e.g., surgical oncology, haematology, radiation oncology, medical oncology, or palliative care. Has links to other services and may provide outreach support

• Primary care: (community setting, general practice etc.)

Table 7 presents the parsimonious linear regression model for predictors of job satisfaction score, adjusting for age and CNSA membership, given the sample characteristics are significantly different to the AHPRA data. Positive Beta (ß) values imply a positive association between variables. Significantly higher scores on job satisfaction were determined by those who “agreed/strongly agreed” they had career progression opportunities (ß 5.64 [2.56–8.73]; *P* < 0.001) and adequate peer support (ß 6.53 [3.29–9.77]; *P* < 0.001), compared to responses in all other categories (“strongly disagree/disagree/neutral”). Conversely, those who “agreed/strongly agreed” they had insufficient resources to provide quality care and lack of leadership had significantly lower scores on job satisfaction, compared to all other categories (ß -4.99 [-7.86- -2.11]; *P* = 0.001 & ß -6.11 [-9.12- -3.11]; *P* < 0.001). Intention to stay in nursing for longer than 5 years (compared to less than 5 years), satisfaction with pay (compared to not satisfied) and using knowledge and skills often or most of the time (compared to none of the time) were also significant predictors of higher job satisfaction score.

**Table 7:** Linear regression model of predictors of job satisfaction score

Variable	Beta [95% CI]	*P* Value
*Have Professional Development Opportunities*		
Strongly disagree/disagree/neutral	Ref	
Agree/strongly agree	3.09 [-0.67–6.85]	0.107
*Have Career Progression Opportunities*		
Strongly disagree/disagree/neutral	Ref	
Agree/strongly agree	5.64 [2.56–8.73]	< 0.001
*Have adequate peer support*		
Strongly disagree/disagree/neutral	Ref	
Agree/strongly agree	6.53 [3.29–9.77]	< 0.001
*Use of full extent of knowledge and skills*		
None of the time/occasionally	Ref	
Often/Most of the time	13.00 [8.11–17.88]	< 0.001
*Intention to stay in nursing*		
Less than 5 years	Ref	
5–9 years	7.55 [3.73–11.36]	< 0.001
10-19years	11.88 [7.33–16.41]	< 0.001
20 or more years	9.27 [4.43–14.12]	< 0.001
*Satisfaction with pay*		
No	Ref	
Yes	4.89 [2.11–7.87]	0.001
*Have insufficient resources to provide quality care*		
Strongly disagree/disagree/neutral	Ref	
Agree/strongly agree	-4.99 [-7.86- -2.11]	0.001
*Lack leadership*		
Strongly disagree/disagree/neutral	Ref	
Agree/strongly agree	-6.11 [-9.12- -3.10]	< 0.001
*Age group in years*		
20–34	Ref	
35–44	1.53 [-2.63–5.69]	0.471
45–54	0.38 [-4.33–5.10]	0.873
55–64	10.01 [5.32–14.80]	< 0.001
65 +	25.05 [17.42–32.67]	< 0.001
*CNSA Member*		
No	Ref	
Yes	0.76 [-2.11–3.63]	0.602

*N* = 635; *P* = < 0.001 (overall model); Adjusted R-squared = 0.362

## Discussion

This analysis of the Cancer Nursing Workforce Mapping project aimed to understand who and where cancer nurses in Australia are and determine the predictors of job satisfaction. Our findings highlight that Australian cancer nurses are highly qualified and experienced, worryingly though a substantial percentage (40–60%) intend to stay in the profession less than 10 years with nurses who were less satisfied in the workplace indicting they were more likely to leave. This is concerning because well-trained cancer nurses are pivotal to the provision of high-quality care, and there must be an adequate number to meet the needs of the patient population [8]. This includes not only the number of nurses, but also the skills and qualifications they possess, the work environment they are in, and their ability to effectively work with the multidisciplinary team [29]. It is of particular importance to retain nurses aged under 50 years who have significant contributions to make to the workforce. Workforce shortages lead to increased stress, burnout and dissatisfaction, further exacerbating workforce problems [16, 30]. Consequently, addressing this shortage is a crucial component to improve job satisfaction and the overall well-being of nurses.

We identified important contributors to job satisfaction at the individual, organizational and systems levels. At the individual level, high workloads were the most reported challenge. Dissatisfaction with workload has markedly increased since the last analysis of pressures in Australia (undertaken over two decades previously) [31], which reported 33% of nurses were dissatisfied with workload compared to the 88% in this current study. Increased workload that has evolved over time can be attributed to multiple factors including the recognised staff shortages, increased patient acuity with rises in chronic disease, and a surge in administrative responsibilities. The integration of technology in healthcare has also changed the way care is delivered and recorded, adding new layers of complexity to nursing responsibilities. A recent study in the UK reported nurses felt dissatisfied and demoralised when they missed care due to high workload and were unsupported when concerns were raised [32].

At the individual level, we found job satisfaction tended to rise with age. Older nurses were more likely to be satisfied with their work. However, these nurses also expressed their intention to leave the workforce as they approach retirement age. Nurses with either less than 5 or more than 20 years’ experience reported higher job satisfaction compared to their mid-career counterparts. Those in senior roles were more likely to be satisfied compared to those working as bedside nurses. The importance of career progression opportunities is highlighted; as nurses’ skills, knowledge and experience develops over time, so too does the expectation of opportunities to advance in their career. Strategies to raise the profile of nursing include linking the knowledge, skills and attributes of nurses to nursing-sensitive patient outcomes [33]. This may be realised by incorporating metrics to measure nursing sensitive outcomes, such as outcomes from nurse-led clinics, into routine reporting.

At an organizational level, our findings highlight the effects of culture in the workplace, such as communication norms, leadership styles and team dynamics. Addressing the problem of high workload should be a priority of organisations. Additionally, lack of leadership, poor peer support, and poorly defined roles were contributors to lower job satisfaction. Conversely, career progression and professional development opportunities were predictors of higher job satisfaction. A recent review identified high job demand, lack of control, lack of social support and lack of recognition were linked to low levels of job satisfaction [3]. Heavy workloads are a major cause of dissatisfaction and can result in high staff turnover. Evidence-based strategies to address this include adopting a teams-focussed approach to improve teamwork [34], cross-training and rotating rosters to ensure appropriate resourcing and staffing levels to reduce inefficiencies [35]. Opportunities for education, training, mentorship, career advancement, and the ability to work to the top of scope of practice are integral to improve job satisfaction [2]. Recognition of the integral role of nurses from senior management can also improve workplace culture and job satisfaction [29]. Staff wellness programs have also been successful at addressing workplace stress [36]. Additionally positive work environments that value nurses in leadership positions, ensure their voices are heard, and respect their ability to advocate for high-quality patient care are understood fosters positive relationship between teams [37]. Addressing the wide variation in nomenclature for the title of nursing roles is pivotal to improve understanding, clarity, and expectation of different roles.

At a systems level, job satisfaction is associated with remuneration. Our study reported pay rates are not equanimous across Australia with nurses in some states receiving higher award rates of pay for the same role compared to other states. Having enough resources to provide quality care also predicted higher job satisfaction. Australia is a large nation with a relatively small population geographically scattered across the land mass. Almost 40% of the population lives in regional or remote parts of the country, which makes the organisation of health services complex. Despite the role of telehealth and other innovative strategies to improve cancer services in rural areas, numerous barriers remain including appropriate governance [38]. Strategies to address these issues include a review of recruitment and retention processes- ensuring that competitive salaries, benefits and professional development opportunities are offered [39]. It is also imperative to identify ways to ensure the knowledge of senior cancer nurses is not lost as a critical mass of the workforce ages into retirement. Succession planning, job shadowing, mentoring and encouraging participation in professional organisations are strategies to ensure knowledge is not lost [29]. It is important the cancer nurses’ voice, no matter their level, are actively involved in any research undertaken around the role and workforce issues. In this way, cancer nurses can contribute more broadly to solving workforce issues at systems and organisational levels and contribute to informing health policy. With a large proportion of senior nurses approaching retirement, documenting their experiences is also critical to help to preserve their knowledge.

These findings are valuable for government/partnership opportunities and policy development. This information will enable the CNSA to better represent cancer nurses across Australia to inform future directions, expansion, and advocacy of the workforce, ensuring cancer nurses have an active seat at the table at the policy level. The findings from this study can also be used as a reference point for future research and will help in making informed decisions on how to support and improve the Cancer nursing workforce in Australia.

### Strengths and limitations

As a cross-sectional study, our research has certain limitations that must be acknowledged including selection bias. Our response rate is estimated at 34% of CNSA members and 13% of all cancer nurses affecting generalisability. Indeed, we identified respondents to the CNSA survey differed in characteristics from the data available from AHPRA regarding cancer nurse demographics. Overall, these findings suggest that the CNSA sample may be an older, more experienced segment of the cancer nursing workforce and may have different characteristics and experiences compared to the broader population of cancer nurses in Australia. We did however control for CNSA membership and age in our analyses of job satisfaction to mitigate this limitation.

We were not able to detect temporal changes over time. This further highlights the importance of understanding and documenting the issues identified in this study for future reference. Our findings may not be representative of cancer nurses from other nations, with different health service funding models, cultures, and opportunities for professional development.

Strengths of this study include our comparison with national registration data, allowing readers to determine generalisability to their specific setting. Additionally, we had a relatively large sample size of respondents which increases the confidence in our findings.

### Implication for practice

Workforce issues are highlighted as priorities for cancer nursing research [40, 41], and this study contributes to the scant evidence base, raising awareness of the factors that contribute to job satisfaction, which may positively influence retention in the workforce. Findings may be used at the individual, service, and systems level to advocate for greater recognition of the contribution of cancer nurses in health policy.

We have highlighted workplace factors that contribute to lower job satisfaction; understanding these can be used to develop strategies, and to identify opportunities for growth and sustainability in the workforce. Further research is required to describe and evaluate the changing scope of nursing practice and roles and effects on patient outcomes [42]. It is also critical to explore strategies to retain the wealth of knowledge in the ageing workforce who have signalled their intention to leave. The next 10 years provide a window of opportunity to harness knowledge and experience and to embed sustainable ways to share this with new generations and future leaders in cancer nursing [29].

## Conclusion

Cancer nurses are critical to the delivery of cancer care however, the workforce is challenged with shortages. This study provides an understanding of the Australian cancer nursing workforce characteristics, their roles and activities, and highlights important considerations for retaining nurses in the profession. We identified individual, organizational, and systemic factors that contribute to job satisfaction including workload, lack of leadership, poor peer support, lack of clearly defined roles, and opportunities for education and career advancement. Strategies to address these are discussed including valuing nurses as leaders in health care and policy. Findings can be used to address and prioritize workforce challenges.

### Supplementary Information

**Additional file 1.**

## Data Availability

The datasets generated and/or analysed during the current study are not publicly available due to ethical approval restrictions but are available from the corresponding author on reasonable request and proof of ethical approval.

## Abbreviations

CNSA: Cancer Nurses Society of Australia
AHPRA: Australian Health Practitioners Regulation Agency
