# Supplementary material for: The cancer nursing workforce in Australia: a national survey exploring determinants of job satisfaction
Source: BMC Nurs. 2023 Dec 6;22:461. doi: 10.1186/s12912-023-01629-7 (PMC10698984; doi:10.1186/s12912-023-01629-7)
Supplement: Supplementary file 1 — Additional file 1. [file 12912_2023_1629_MOESM1_ESM.pdf]

Cancer Nursing Workforce Survey

PARTICIPANT INFORMATION FOR RESEARCH PROJECT

- for identifying cancer nursing workforce in Australia -

Cancer Nursing Workforce Australia

Description

To date, there has been no study undertaken on the oncology nursing workforce across Australia.

Participation

Participation in the project is entirely voluntary. Participation involves completing an anonymous online survey about your views on cancer nursing workforce. You will also be asked some questions about yourself (e.g., your age group) and your role in cancer nursing. The survey will take approximately ten (10) minutes of your time.

If you do agree to participate, you can withdraw from the project at any time without comment or penalty by not completing the survey or stopping completion of the survey. Your decision to participate, or not participate, will in no way impact upon your current or future relationship with CNSA. If you agree to participate, you do not have to complete any questions that you are uncomfortable answering.

If you decide to participate in this study, please complete the survey via the available link at the bottom of this page.

After the study is complete, the results will be made available to CNSA members to guide future workforce planning and advocacy for cancer nursing in Australia. The results may also be made available to external health professionals through journals and meetings. Individual participants (including yourself) will not be identifiable in any of these reports.

Expected benefits and risks

It is expected that there will be no immediate benefit to you for your participation in the survey. However, it is anticipated that the outcomes of this survey will identify current cancer nursing workforce challenges and opportunities identified by cancer nurses in Australia and may lead to the development of new knowledge and resources to address the concerns identified.

There are no risks beyond normal day-to-day living associated with your participation in this survey. However, if you feel uncomfortable with any of the questions asked, you do not need to complete the survey.

Privacy and confidentiality

The study will be conducted according to the NHMRC National Statement on Ethical Conduct in Human Research. This statement aims to promote ethical human research and to ensure that participants are afforded the respect and protection that is due to them.

The data obtained from your comments and responses to the survey will be treated confidentially. Your data will remain anonymous as you will not be asked to provide your name or identifying information. As your identity will not be linked to the survey data, any comments or responses you provide will not be able to be withdrawn. Data will be analysed and reported in an aggregate form that it will not be possible to trace it back to you. Data will be stored securely and only authorised persons, who understand it must be kept confidential, will have access to it. Study records will be kept in password protected electronic files at Monash during the study, for at least 5 years from the time the study is closed, and securely destroyed thereafter. Please note that non-identifiable data collected in this project may be used as comparative data in future related projects.

Research team

If you have any questions or require any further information, please contact:

XX is committed to research integrity and the ethical conduct of research projects and this study has received a waiver of need for ethical approval from the XX Human Research Ethics Committee (number xxx). However, if you have any concerns or complaints about the ethical conduct of the project you may contact the XX Research Ethics Officer on XXXX

I have read and understood the study information and consent to participate.

◯ I Agree
◯ I Disagree

Thank you for your participation.

Firstly, please answer these few demographic questions.

Age group (years)

◯ 19-24
◯ 25-29
◯ 30-34
◯ 35-39
◯ 40-44
◯ 45-49
◯ 50-54
◯ 55-59
◯ 60-64
◯ 65-69
◯ 70-74
◯ 75+

Gender

◯ Female
◯ Male
◯ Prefer not to say
◯ Other (non-binary)

What state do you work in?

◯ ACT
◯ QLD
◯ NSW
◯ VIC
◯ SA
◯ WA
◯ NT
◯ TAS
◯ Other (eg Norfolk Island)

Postcode of workplace

____

Years of nursing experience since obtaining nursing qualifications

◯ Less than 5 years
◯ 5-9 years
◯ 10-19 years
◯ 20 years or more

What is your highest qualification?

◯ Hospital General Nursing Certificate
◯ Post Registration Certificate/Diploma
◯ Bachelor Degree
◯ Masters graduate entry to nursing
◯ Postgraduate

Did you obtain your nursing qualifications in Australia?

◯ Yes
◯ No

Which country did you obtain your nursing qualifications in?

____

What type of postgraduate qualification do you have?
(tick all that apply)

☐ Graduate Certificate/Diploma
☐ Masters (Coursework)
☐ Masters (Research)
☐ Professional Doctorate
☐ PhD

Years of cancer nursing experience

◯ Less than 5 years
◯ 5-9 years
◯ 10-19 years
◯ 20 years or more

Do you have a cancer related qualification?

◯ Yes
◯ No

Are you currently studying or planning to commence a cancer related qualification in the next 12 months?

◯ Yes
◯ No

These next questions relate to your nursing role.

What is you main nursing role?

◯ Enrolled Nurse
◯ Registered Nurse
◯ Clinical Nurse
◯ Nurse Coordinator/Navigator
◯ Clinical Nurse Consultant/Specialist/Liaison
◯ Nurse Unit Manager
◯ Nurse Educator
◯ Clinical Trials Nurse
◯ Research Nurse (supporting research led by someone else)
◯ Researcher/Academic (undertaking own research or teaching)
◯ Director of Nursing
◯ Nurse Practitioner
◯ Other (Please specify below)

If "Other" please comment: ____________________

Type of employment

◯ Full-time
◯ Part-time
◯ Casual

How would you describe yourself?

☐ I am a recently graduated nurse (three years or less in the workforce)
☐ I am an experienced nurse new to cancer nursing
☐ I am an experienced nurse working in cancer care
☐ Other

If "Other" please comment: ____________________

Do you provide clinical nursing care to cancer patients?

◯ Yes
◯ No

The EdCAN professional development models described 4 tiers of clinical cancer nursing practice.

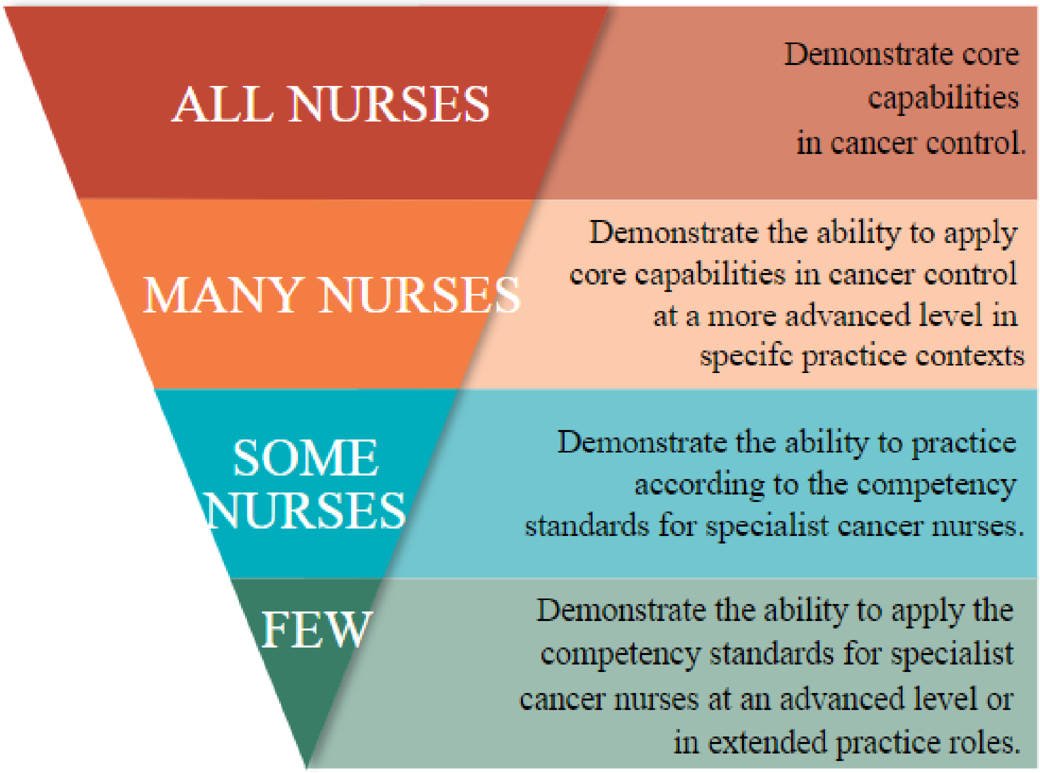

If you would like to view EdCAN competency standards for more information, please open the below attached document

[Attachment: "competency standards.docx"]

Which tier best describes your clinical experience in caring for people affected by cancer?

◯ Tier 1- I care for people affected by cancer infrequently, and can demonstrate core capabilities in cancer care
◯ Tier 2 - I care for people affected by cancer regularly in a non-specialist setting, and can apply core capabilities at a more advanced level in specific context
◯ Tier 3 - I provide specialist cancer care in a dedicated cancer service, adhering to the competency standard for specialist cancer nurses
◯ Tier 4 - I am authorised to practice at an advanced/extended role applying the competency standard for specialist cancer nurses in this role.

Which facility type best describes your workplace
(tick all that apply)

☐ A cancer centre: Provides specialised, multidisciplinary service and specialised interventions to manage common and rare cancers. May provide outreach support.
☐ A cancer unit: Provides a multidisciplinary service to manage most common cancers. May provide outreach support.
☐ A cancer service: May consist of single service e.g. surgical oncology, haematology, radiation oncology, medical oncology or palliative care. Has links to other services and may provide outreach support.
☐ Primary care (community setting, general practice etc)
☐ Other

Which category best describes your work place funding?

- ☐ Private
- ☐ Public
- ☐ Public/Private
- ☐ Not for Profit Organisation

If "Other" please comment: __________________________________

We know there are differences in relation to the level a specialist cancer nurse is employed as, and the different tiers of cancer nursing roles. Please answer the following questions about your current role

What is the title of your role

____

What industrial award/agreement are you employed under

- ☐ Nurses Award- Industrial (applies to primary healthcare and general practice)
- ☐ Public Sector Nurses and Midwives Award
- ☐ Private Sector Industry Nurse State award
- ☐ Other enterprise agreement
- ☐ I am not employed under a nursing award/agreement

What is the main cancer speciality you work in
(tick all that apply)

- ☐ Medical Oncology
- ☐ Haematological Oncology
- ☐ Surgical Oncology
- ☐ Radiation Oncology
- ☐ Palliative Care
- ☐ Paediatric Oncology
- ☐ Adolescent Young Adult Oncology
- ☐ Cancer Coordination
- ☐ Community Oncology
- ☐ Other

If "Other" please comment:

____

What are the main tumor streams you care for

(tick all that apply)

- ☐ Haematology
- ☐ Upper GI
- ☐ Lower GI
- ☐ Lung Cancer
- ☐ Melanoma
- ☐ Brain/CNS
- ☐ Urogenital
- ☐ Prostate
- ☐ Gynaecological
- ☐ Breast
- ☐ Sarcoma
- ☐ Rare cancers (including paediatric)
- ☐ Neuroendocrine
- ☐ Other

If "Other" please comment:

____

What types of activities are you often involved in?

(tick all that apply)

☐ Inpatient care
☐ Outpatient care
☐ Homecare
☐ Chemotherapy / Immunotherapy administration
☐ Supportive care (eg blood transfusions, management of infection, mucositis etc)
☐ Surgical care
☐ Palliative care
☐ Radiotherapy
☐ Patient education
☐ Staff education
☐ Care coordination
☐ Management
☐ Research

What professional organisations are you a member of?
(tick all that apply)

☐ Cancer Nurses Society of Australia (CNSA)
☐ Clinical Oncology Society Australia (COSA)
☐ Palliative Care Nurses Australia (PCNA)
☐ Haematology Society Australia and New Zealand (HSANZ)
☐ Paediatric Palliative Care Australia and New Zealand (PaPCANZ)
☐ International Society of Nurses in Cancer Care (ISNCC)
☐ Australian and New Zealand Children's Haematology Oncology Group (ANZCHOG)
☐ Oncology Nurses Society (ONS)
☐ Australian College of Nursing (ACN)
☐ Other

If "Other" please comment: ______________________

How long do you intend to stay in cancer nursing?

◯ Less than one year
◯ 1-2 years
◯ 3-5 years
◯ 6-10 years
◯ 11-20 years
◯ 20 years +
◯ Other

If "Other" please comment: ______________________

These questions ask about how your knowledge and skills are used in the workforce.
There are no right or wrong answers, please choose the answer than best describe your situation.

How often do you feel you get to use the full extent of your knowledge and skills?

◯ None of the time
◯ Occasionally
◯ Often
◯ Most of the time

These next questions ask about your perceptions of your cancer nursing role.

Some questions relate to clinical roles and may not be applicable to your role.

	Strongly Disagree	Disagree	Neutral	Agree	Strongly Agree	Not applicable
I can confidently provide comprehensive cancer care	○	○	○	○	○	○
I can confidently manage the physical symptoms of patients with cancer	○	○	○	○	○	○
I can confidently manage the psychological symptoms of patients with cancer	○	○	○	○	○	○
I can confidently manage symptoms of spiritual distress in patients with cancer	○	○	○	○	○	○
My role has a clearly defined scope of practice	○	○	○	○	○	○
I have opportunities for professional development in my workplace	○	○	○	○	○	○
I participate in multidisciplinary discussions about the care of patients	○	○	○	○	○	○
I am able to make autonomous decisions about the cancer nursing care I deliver to patients	○	○	○	○	○	○
I receive the educational support I need from my organisation to undertake my role	○	○	○	○	○	○
I receive adequate peer support in my workplace to undertake my role	○	○	○	○	○	○
There are opportunities for me to progress my career	○	○	○	○	○	○

What would you like to do more often in your cancer nursing role?

What are the challenges to the cancer nursing workforce in your workplace?

	Strongly Disagree	Disagree	Neutral	Agree	Strongly Agree
Managing high workload	☐	☐	☐	☐	☐
Information overload	☐	☐	☐	☐	☐
Lack of clarity about roles/ performance expectations	☐	☐	☐	☐	☐
Lack of opportunities for career progression	☐	☐	☐	☐	☐
Lack of education and training opportunities	☐	☐	☐	☐	☐
Poor clinical supervision or mentorship	☐	☐	☐	☐	☐
Lack of leadership in the workplace to support workforce	☐	☐	☐	☐	☐
Insufficent resources to provide quality care	☐	☐	☐	☐	☐
Low motivation of staff to provide quality care	☐	☐	☐	☐	☐
Ineffective interagency collaboration	☐	☐	☐	☐	☐
Integrating digital health technologies e.g. telehealth, electronic medical records	☐	☐	☐	☐	☐

How satisfied are you with your current job?

Very dissatisfied   Neutral   Very satisfied

(Place a mark on the scale above)

Please comment on your job satisfaction

____

What opportunities or initiatives would you like to see developed to support the cancer nursing workforce in Australia?

____

What suggestions do you have for improvement for the cancer nursing workforce in your workplace?

____

Where do you see cancer nursing in 10 years time?

____

Please tell us about any other workforce issues you believe are important to acknowledge.
For example, levels of emotional fatigue, depersonalisation, personal accomplishment and satisfaction with cancer nursing career.

If you are interested in participating in an interview to determine key workforce challenges and recommendations for improvement please provide your email address so we can contact you. Your email address will be registered separately to the survey, so your survey responses remain anonymous. __________________________________

Thank you for your participation! We value your contribution. Have a lovely day.
